# Regeneration and Long-Term Stability of a Low-Power Eco-Friendly Temperature Sensor Based on a Hydrogel Nanocomposite

**DOI:** 10.3390/nano14030283

**Published:** 2024-01-30

**Authors:** Giovanni Landi, Sergio Pagano, Veronica Granata, Guerino Avallone, Luca La Notte, Alessandro Lorenzo Palma, Paolo Sdringola, Giovanni Puglisi, Carlo Barone

**Affiliations:** 1 ENEA, Portici Research Center, Piazzale Enrico Fermi, Località Granatello, 80055 Portici, Italy; giovanni.landi@enea.it; 2 Dipartimento di Fisica “E.R. Caianiello”, Università degli Studi di Salerno, 84084 Fisciano, Italy; vgranata@unisa.it (V.G.); guavallone@unisa.it (G.A.); 3 INFN Gruppo Collegato di Salerno, Università degli Studi di Salerno, 84084 Fisciano, Italy; 4 CNR-SPIN, Università degli Studi di Salerno, 84084 Fisciano, Italy; 5 ENEA, Casaccia Research Center, Via Anguillarese 301, 00123 Rome, Italy; luca.lanotte@enea.it (L.L.N.); alessandrolorenzo.palma@enea.it (A.L.P.); paolo.sdringola@enea.it (P.S.); giovanni.puglisi@enea.it (G.P.)

**Keywords:** temperature sensor, sustainability, environmental monitoring, gel polymer electrolyte, gelatin, graphene, water processable, self-powered, current limiting phenomena, faradaic process, energy efficiency

## Abstract

A water-processable and low-cost nanocomposite material, based on gelatin and graphene, has been used to fabricate an environmentally friendly temperature sensor. Demonstrating a temperature-dependent open-circuit voltage between 260 and 310 K, the sensor effectively detects subzero ice formation. Notably, it maintains a constant temperature sensitivity of approximately −19 mV/K over two years, showcasing long-term stability. Experimental evidence demonstrates the efficient regeneration of aged sensors by injecting a few drops of water at a temperature higher than the gelation point of the hydrogel nanocomposite. The real-time monitoring of the electrical characteristics during the hydration reveals the initiation of the regeneration process at the gelation point (~306 K), resulting in a more conductive nanocomposite. These findings, together with a fast response and low power consumption in the range of microwatts, underscore the potential of the eco-friendly sensor for diverse practical applications in temperature monitoring and environmental sensing. Furthermore, the successful regeneration process significantly enhances its sustainability and reusability, making a valuable contribution to environmentally conscious technologies.

## 1. Introduction

Natural biopolymer hydrogels have attracted considerable interest owing to their distinctive qualities, including eco-friendliness, high water affinity, biocompatibility, flexibility, and customizable physical and chemical properties [1,2,3]. These characteristics have prominently positioned them in various applications spanning from the pharmaceutical to biomedical sectors, playing a crucial role in drug delivery mechanisms [4,5,6] and tissue engineering [7,8].

In recent decades, the integration of natural biopolymer hydrogels with various fillers, such as activated carbon, graphene, and graphene oxide, has given rise to hydrogel nanocomposites [2,9,10,11,12,13]. These composites have gained widespread use as smart materials in the field of electronics, contributing significantly to advancements in sensing technologies [9,10,11]. Moreover, hydrogel nanocomposites have demonstrated relevance also in the field of energy storage devices [2,12,13]. More recently, substantial attention has been directed towards hydrogel-based electrolytes, propelled by their ionic conductive properties and their capacity to establish a galvanic cell with metal electrodes [14,15,16,17]. Jonsson et al. reported a self-powered temperature sensor assisted by electrolyte (operating within a 15 °C variation), utilizing a hydrogel with a voltage sensitivity of approximately −11 mV/K [15]. Here, the sensor is based on two separated gold (Au) and aluminum (Al) electrodes connected via an electrolyte. Ortega et al. detailed the development of a self-powered intelligent patch designed for the monitoring of sweat conductivity, wherein the bodily fluid functions as the electrolyte for the device battery [16]. Moreover, Zhao et al. developed a sodium alginate-based composite film for self-powered humidity sensing with a power density of 0.5 μW cm^−2^. Integrated with a capacitor and a LED (light-emitting diode), the system indicates different humidity levels through LED brightness [17]. In this context, the advancement of sensors with minimal or zero energy consumption plays a pivotal role in promoting sustainable energy management and facilitating the transition away from fossil fuels [18]. Furthermore, the utilization of natural biomaterials, including chitosan, gelatin, and casein derived from renewable resources as sustainable smart materials for electronics applications, serves to diminish the environmental impact and enhance sustainability [1,9,10,11,19,20]. This approach represents a valuable alternative to the potential escalation of electronic waste resulting from the widespread proliferation of the Internet of Things (IoT), which could have adverse effects on both humans and the environment [21].

Under these premises, a recently developed eco-friendly temperature sensor utilizes a cost-effective water-processable nanocomposite of gelatin and graphene. This sensor, with a temperature sensitivity of approximately −19 mV/K, demonstrates the capability to detect ice formation at a few μA. Operating within the temperature range of 260 K to 310 K, it offers a fast response, low power consumption, long-term stability, and easy disposability, rendering it highly suitable for environmental indoor monitoring [9]. Recently, cement-based nanocomposites based on carbonaceous fillers (e.g., carbon black, graphene, and graphene oxide) have been extensively applied to enhance the mechanical properties, durability, and self-sensing performance of construction materials [22,23]. Gelatin, a biodegradable polymer obtained from collagen hydrolysis, forms hydrogels with enhanced dielectric and mechanical properties due to its ability to trap liquid molecules and fillers [20,24,25]. The prevalence of polar functional groups in the gelatin hydrogel facilitates the development of various applications, including actuators [26,27,28,29], sensors [10,30,31,32], and organic transistors [33,34,35].

In the present study, an evaluation of the long-term stability of the temperature sensor, based on a hydrogel–gelatin nanocomposite, has been conducted over two years. This evaluation has been compared in terms of speed response, energy consumption, and power consumption with the sensor parameters in pristine conditions. Similar to its initial state, the aged device exhibited limiting-current phenomena in the gelatin electrolyte, attributed to dissociation reactions. The proposed model underscored distinctions in charge carrier accumulation, faradaic charge transfer, and diffusion processes within the device in its pristine and aged state under the current biasing.

Additionally, the aged temperature sensor maintains a low power consumption in the microwatt range, with approximately 30% of its power being self-generated by the galvanic cell formed between the electrode interfaces. Furthermore, the thermal stability of the gel nanocomposite has been evaluated within the temperature range of 297 K to 323 K. Experimental evidence substantiates the successful regeneration of the aged temperature sensor through water absorption at a temperature surpassing the gelation point of the hydrogel nanocomposite.

## 2. Materials and Methods

**Figure 1:**
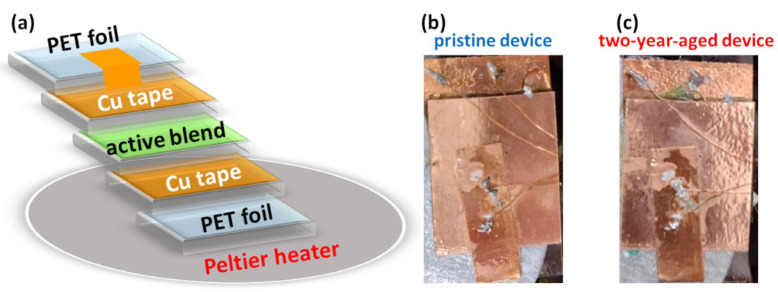
(**a**) Cross-section of the symmetric device. Top view photographs of typical investigated pristine (**b**) and two-year-aged (**c**) devices, respectively.

The investigated devices were realized by sandwiching in a symmetric way, as shown in Figure 1a, an active nanocomposite between plastic substrates covered with copper (Cu) tape as electrodes. In detail, the active blend was prepared by dissolving gelatin extracted from bovine skin (type B), having a gel strength of about 225 g Bloom, in a mixture of Milli-Q water (18.2 MΩ cm) and glycerol (purchased from Sigma-Aldrich, Milano, Italy) at 80 °C. This aqueous gelatin solution, having a concentration of 10 wt.%, was subsequently enriched with graphene nanoplatelets (GNPs) (purchased from Sigma-Aldrich, Milano, Italy), in order to form mixtures with 0.25 wt% of filler contents. A mechanical stirring process was then performed for about 30 min at the same temperature of 80 °C. The obtained gel nanocomposite was deposited by blade coating on a substrate of polyethylene terephthalate (PET) foil (Melinex ST 504, DuPont Teijin Films, Chester, VA, USA, thickness 125 μm), covered with Cu tape (Kohree, City of Industry, CA, USA, thickness 40 μm), and dried at room temperature. The thickness of the active layer is approximately 1800 µm. The gelatin/graphene system exhibits a layered and homogeneous morphology, providing evidence of the well-dispersed filler [20,24]. All the details on fabrication, morphological, and electrochemical characterizations are reported elsewhere [9]. The sandwich structure was completed with a top electrode of PET-Cu foil, having an area of 2.5 × 2 cm^2^.

The experimental electric transport investigations, performed on pristine (Figure 1b) and two-year-aged devices (Figure 1c), explored a wide temperature region from 250 to 340 K, which is the typical operating region of environmentally friendly temperature sensors. A thermoelectric-cooled Peltier-type system was used to vary the temperature, measured with a LM35 sensor (Texas Instruments, Dallas, TX, USA) in contact with the sample holder and stabilized through a computer-controlled Proportional-Integral-Derivative (PID) loop to better than 0.2 K. A low-noise Keithley dc current source (Tektronix Inc., Beaverton, OR, USA) and a digital multimeter were adopted to bias the samples and to record the output dc voltage drop. All the bias and readout circuitry was connected through a low-noise homemade electronics [36], already successfully used for the measurement of photovoltaic devices [37].

## 3. Results

The incorporation of a small amount of graphene as a filler enhances the dielectric properties of the active material, surpassing those of pure gelatin and achieving an electrode-specific capacitance value of approximately 380 F/g [20]. It is noteworthy that utilizing a minimal amount of graphene filler, specifically below the percolation threshold, serves to prevent the formation of a conductive network within the composite system. In contrast, the sample containing 1 wt% of graphene exhibits a decline in dielectric properties coupled with an augmentation in electrical conductivity [20]. Notably, the conductive filler significantly influences the reaction kinetics between the electrode and the biopolymer [24]. To ensure an environmentally safe and easily disposable device, any ionic components potentially detrimental to the environment have been avoided. In this context, gelatin serves as a binder, holding together the hydrophobic filler with water–glycerol molecules and acting as a solid electrolyte, functioning as a protonic conductor [38].

The fabricated device works as a temperature sensor, detecting variations in temperature (∆T) with a negative dependence. This characteristic is quantified by the voltage sensitivity $m_{V}=∆V/∆T$, where ∆V represents the change in the output electrical signal. Several authors have reported the existence of temperature sensors derived from natural biopolymers sourced from renewable materials [1,9,15,39]. These biopolymers, including cellulose, silk, chitosan, and even tobacco, have been utilized in the development of sensors that are electrically biased or exhibit self-powering capabilities. Notably, these sensors demonstrate a negative voltage sensitivity in response to temperature variations.

### 3.1. Evidence of the Limiting Current Phenomena in the Pristine and Aged Hydrogel Nanocomposites

**Figure 2:**
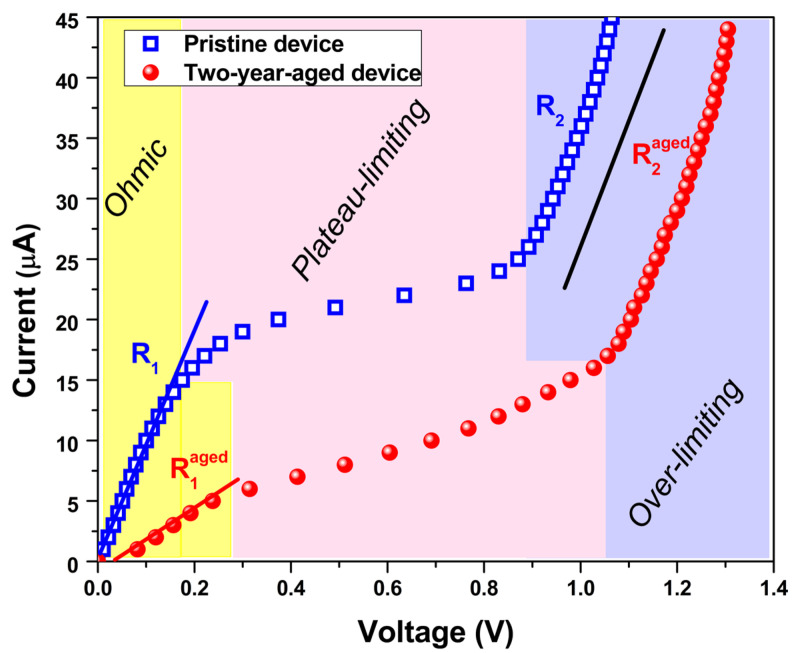
Current–voltage characteristics of the pristine and two-year-aged temperature sensor under current biasing. The colored regions are shifted following the different working conditions of pristine and aged device.

Figure 1b,c depicts images of the device before experimental characterization in its pristine and two-year-aged states, respectively. Given that the temperature sensor in its pristine state exhibits a limiting current phenomenon, a chronopotentiometric characterization for the aged device has been conducted under the same conditions [9,40]. Specifically, a current profile has been imposed, consisting of a series of step impulses with a uniform magnitude (1 μA) and equidistant time intervals (60 s) [9]. As evidenced in Figure 2, the corresponding current–voltage characteristics for both pristine and aged states exhibit three distinct regions identified as ohmic, plateau-limiting, and over-limiting. In the first region, the voltage signal increases linearly in response to the applied current, demonstrating a resistance of 10.3 kΩ ($R_{1}$) and 38.5 kΩ ($R_{1}^{aged}$) for the fresh and aged states, respectively. As expected, the aged device displays greater resistivity compared to the fresh sample, indicating an increase of $R_{1}^{aged}$ that can be attributed to the diffusion of the water molecules out from the blend. This phenomenon has previously been observed in several materials based on gelatin nanocomposites [24]. The introduction of a hydrophobic filler, graphene nanoplatelets, to the biopolymer alters the network formation of protein chains due to limited interaction with the hydrogel. This effect contributes to the diffusion of water–glycerol molecules out of the active blend throughout the two-year ageing period, resulting in increased mechanical stiffness and electrical resistivity [24].

In the plateau-limiting region of the pristine device, the current remains relatively constant while the voltage increases. This specific value denotes the limiting current ($I_{lim}$) at which the dissociation reaction within the device initiates [41]. Given that the nanocomposite is fabricated in a water solution, the resulted blend contains a substantial quantity of water–glycerol molecules confined within [24]. These molecules actively participate in the dissociation processes when the applied bias current ($I_{bias}$) exceeds $I_{lim}$. The ensuing reactions lead to an increase in ion concentration, justifying the observed decrease in resistance ($R_{2}$≈ 6.8 kΩ) measured in the over-limiting region. This behavior is also evident in the aged device, where a similar value of $R_{2}^{aged}$ is observed. To identify the value of $I_{lim}$ for the aged device, the Cowan–Brown method has been applied [42].

Figure S1 (Supplementary Materials) shows the comparison of the Cowan–Brown plots for the pristine and two-year-aged devices. The results indicate that the $I_{lim}$ of the temperature sensor decreases from 13 µA to 5 µA in the aged state, aligning with expectations and indicating that the rise in resistivity ascribed to the loss of water within the blend also impacts $I_{lim}$.

In this context, gelatin serves as a protonic-conducting gel, wherein the diffusion coefficient for H^+^ ions facilitated by hydrogen bonding interactions between the amine and hydroxyl groups of the matrix, as well as water molecules entrapped within the blend [38]. This contrasts with less diffusion characteristics observed for divalent ions, such as Ca^2+^, Cu^2+^, and Fe^2+^ [43]. The essential function of the hydrogen bond network within the gelatin matrix is to facilitate the long-range hopping motion of H^+^ ions (e.g., Grötthus-type mechanism) [44]. This motion is activation-energy-driven and, as a result, is highly sensitive to variations in temperature for several thermoresponsive hydrogels [11,45].

It should be noted that the gelatin and water–glycerol molecules are also mixed homogeneously, contributing to the formation of this ion-conducting path. By increasing the temperature, the number of H-bonds within the biopolymer increases, leading to a variation in the charge carrier accumulation at the metal/gel–nanocomposite interface [9]. The lower quantity of water–glycerol molecules in the aged device causes a notable reduction in the ion-conducting path. Consequently, this produces an increase in resistivity and a concomitant decrease in $I_{lim}$, attributable to the diminished concentration of available water–glycerol molecules participating in the dissociation reactions [11,46]. This phenomenon accounts for the observed shift in the electrical characteristic of the aged device towards the low-current region, as depicted in Figure 2.

**Figure 3:**
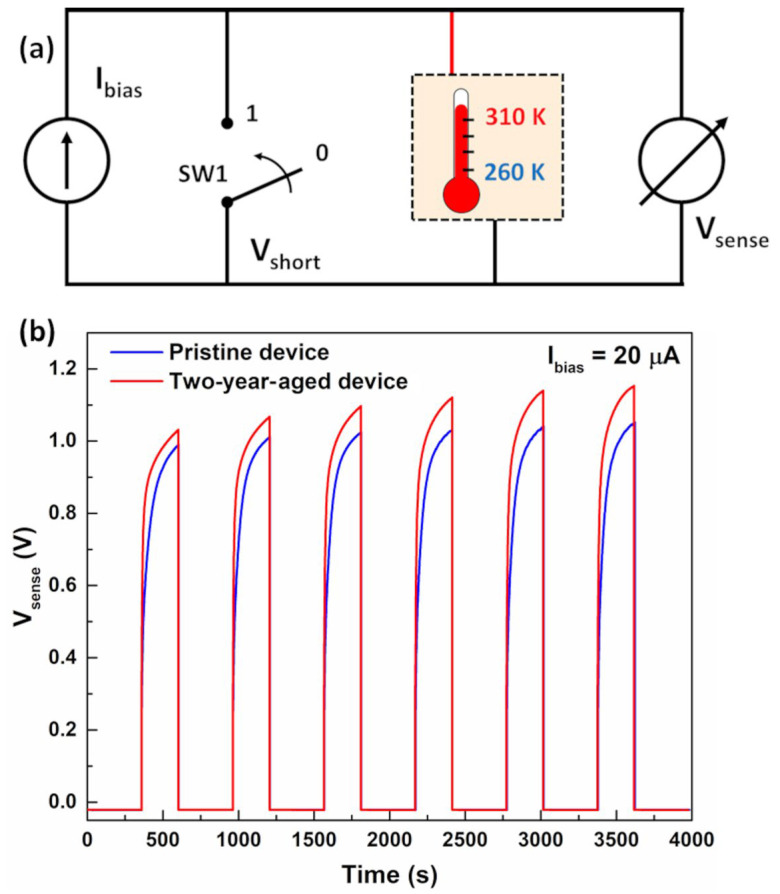
(**a**) Schematic representation of the driving circuit employed for the measurement. (**b**) Time evolutions of the voltage variations across the device in both pristine and aged state, under a bias current of 20 μA at 300 K.

As reported in the literature, the electrochemical temperature sensor, under operating conditions ($I_{bias}\neq 0$), exhibits a voltage drift and offset of the output signal [9]. Moreover, in the off state ($I_{bias}=0$), the output voltage is subject to unregulated self-discharge processes within the device, influenced by the history of the device (e.g., electrode potentials and charges stored before discharge) and measurement conditions (e.g., operating time and bias currents). This results in a memory effect during each measurement cycle, characterized by variations in offset values and introducing inaccuracies in temperature measurements. To address this, a simple bias circuit has been implemented, as depicted in Figure 3a.

When the sensor is turned off, the voltage across the device is electrically shorted using switch ${SW}_{1}$ controlled by the signal $V_{short}$. Under these conditions, the sensor rapidly depletes accumulated charge, achieving a reproducible idle state. Subsequently, upon opening switch ${SW}_{1}$, the bias current flows through the sensor, transitioning the device into sensing mode, causing the voltage $V_{sense}$ to increase until it reaches a saturation value ($V_{sat}$). The circuit design enables a measurement every 10 min, comprising a 6-min idle time and a 4-min sense period ($\tau_{sense}$). The corresponding voltage responses from six consecutive measurement cycles at 300 K under a bias current of 20 μA, representing both pristine and aged states, are illustrated in Figure 3b. As evident, the sensor recovers its open-circuit voltage after being electrically shorted during the idle time. The utilization of this circuit mitigates unregulated discharge phenomena, enhancing the reproducibility of the response in sensing mode. The drift effect diminishes, and the saturation level of the output voltage remains more stable during cycling measurements.

In accordance with the literature findings, the limiting current demonstrates a temperature-dependent relationship, impacting the sensor response. With an increase in temperature (denoted as T), there is a concurrent rise in the limiting current, causing a shift of the plateau region towards higher voltages. This effect is attributed to the heightened ionic conductivity of the blend, stemming from an increase in the concentration of protons released by COOH and NH_3_^+^ groups as T increases [9]. Consequently, the $I_{lim}$ value, directly proportional to the concentration of ions participating in dissociation reactions, undergoes a corresponding increase. This observed trend aligns closely with documented findings in the literature [11,46].

Furthermore, the speed response of the sensor is subject to the influence of the bias current $I_{bias}$. For values below $I_{lim}$, the device works in ohmic regime and manifests a sluggish dynamic response lasting tens of seconds. Conversely, when the system operates with $I_{bias}$> $I_{lim}$, the transients occur at an accelerated rate. This signifies that the processes involving the formation of the double layer at the electrode interface and faradaic charge transfer mechanisms significantly alter the performance characteristics (e.g., speed, sensitivity, linearity) of the electrochemical sensor.

### 3.2. DC Electrical Model of the Electrochemical Temperature Sensor

**Figure 4:**
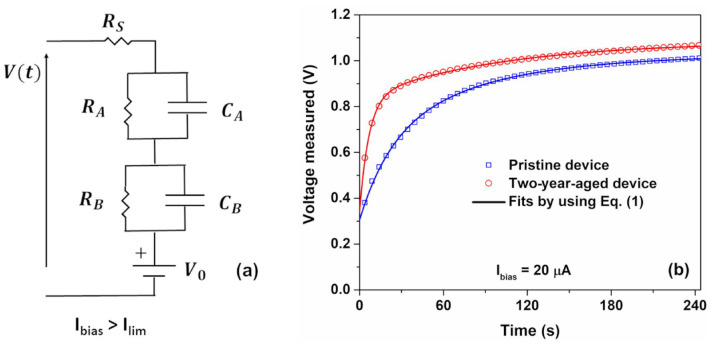
(**a**) DC circuit for the temperature sensor under current-controlled mode for $I_{bias}>I_{lim}$ and (**b**) time evolution of the voltage measured across the device under operating conditions at 300 K and 20 µA for the pristine and aged device, respectively. The best fitting curves, shown as solid lines, are obtained from Equation (1).

The DC electrical model of the electrochemical temperature sensor comprises a series of connections involving resistances that characterize the ohmic aspects of the blend, along with capacitive components originating from the electrode interfaces. The interfaces of the metal/gel nanocomposite are envisioned as capacitances, denoted as *C_A_* and *C_B_*, representing the accumulation of ions at an electrical double layer. The presence of faradaic currents signifies electron transfer through redox reactions at the electrodes. This additional element is modeled as parallel resistances, labeled *R_A_* and *R_B_*, in conjunction with double-layer capacitances (*C_A_* and *C_B_*) [47]. As the aged device exhibits a more resistive nature compared to its pristine state, as depicted in Figure 2, a comprehensive model has been included, incorporating the consideration of faradaic reactions at the cathode. In Figure 4a, a DC circuit for the temperature sensor under current-controlled mode for $I_{bias}>I_{lim}$ is reported. The electrode potentials resulting from the oxidation reaction at the anode and the reduction reaction within the blend proximate to the cathode are denoted as *V_A_* and *V_B_*, respectively [48]. In this context, *V*_0_ *= V_B_ – V_A_* represents the open circuit potential, while *R_S_* encompasses both the ohmic contribution of the space charge layer of the blend and that of the electrodes. The output voltage *V(t)* under bias current is expressed as [9]:

(1) $$V\left(t\right)=V_{0}+R_{S}I_{bias}+R_{A}I_{bias}\left(1-e^{-\frac{t}{\tau_{A}}}\right)+R_{B}I_{bias}\left(1-e^{-\frac{t}{\tau_{B}}}\right),$$

where  t represents time in seconds and *τ_B_ = R_B_ C_B_* and *τ_A_ = R_A_ C_A_* are the time constants linked to the cathode and anode kinetics, respectively. Notably, *τ_A_* plays a fundamental role in influencing the speed response ∆τ of the fresh sensor with values ranging between a few seconds to tens of seconds [9]. It is worth noting that for the pristine device, the cathode functions as an inert electrode, with no faradaic reactions occurring at the interface. Consequently, the parallel resistance *R_B_* is significantly larger than 1, and as a result, the latter term in Equation (1) can be approximated as $\frac{I_{bias}}{C_{B}}t$ [9]. Once a sufficiently extended period $∆\tau \approx 4\tau_{A}$ has elapsed, the voltage response of the sensor attains a saturation level denoted as $V_{sat}\approx V_{0}+I_{bias}\left(R_{S}+R_{A}\right)$ [9]. Consequently, the voltage sensitivity $m_{V}=∆V/∆T$ of the sensor can be expressed as:

(2) $$m_{V}=\frac{{∆V}_{0}}{∆T}+I_{bias}\left(\frac{{∆R}_{S}}{∆T}+\frac{{∆R}_{A}}{∆T}\right),$$

where $m_{o}=\frac{{∆V}_{0}}{∆T}$ is the temperature variation of the open-circuit voltage and $m_{R}=\left(\frac{{∆R}_{S}}{∆T}+\frac{{∆R}_{A}}{∆T}\right)$ is the temperature variation of the resistances associated with the faradaic processes and space charge layer under current biasing, respectively. It is evident that the voltage sensitivity encompasses two contributions: the first pertaining to the self-power component ($m_{0}$) and the second induced by the current biasing ($m_{R}$). It should be noted that the device behaves as a galvanic cell when the $I_{bias}$ is equal to zero. The observed open-circuit voltage (*V_0_*) is attributed to temperature-induced modifications affecting the redox activity, as detailed in reference [9].

The theoretical model represented by Equation (1) has been employed for fitting the experimental data, illustrated by solid curves in Figure 4b. The resultant best-fitting parameters are detailed in Table 1. It is evident that over the two-year time span, the aged device exhibits an increase in resistance contributions (*R_S_* and *R_A_*), attributable to the drying process of the gelatin nanocomposite, in comparison to its initial state. Notably, the ageing phenomenon alters the cathode behavior, leading to a reduction in the parallel resistance *R_B_* from approximately 108 kΩ to 45 kΩ for the aged device. This signifies that, under operational conditions, the cathode electrode does not act as an inert electrode and starts to contribute to the electrical response under bias current. The occurrence of faradaic reactions at the cathode interface induces modifications in the electrode/gel nanocomposite interface, as evidenced by the rise in the *C_B_* parameter, which models the charge accumulation of the electrode.

Nevertheless, the speed response of the temperature sensor appears unaffected by these ageing phenomena, as indicated by similar values of ∆τ for both device states. Similarly, the self-powered contribution, modeled by the *V*_0_ parameter, remains unaffected. The existence of a galvanic cell at the metal/gel nanocomposite interface facilitates a reduction in power consumption for the device, mediated by the electrode potential *V*_0_. Consequently, the sensor manifests a partial self-powering capability, generating approximately 30% of the requisite energy for its operational needs, as listed in Table 1. Furthermore, the voltage sensitivity $m_{V}$, described in Equation (2), also incorporates the contribution related to self-powering.

### 3.3. Temperature Dependence of the Electrical Characteristics

**Figure 5:**
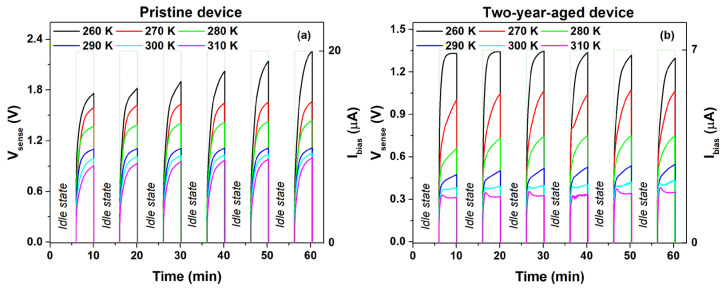
Temperature-dependent profiles illustrating the temporal evolution of voltage across the device in both the (**a**) pristine and (**b**) aged states, under bias currents of 20 μA and 7 μA, respectively. The measurements have been conducted utilizing a logic circuit.

Due to the increased resistive contribution observed in the aged device, the bias current value ($I_{bias}>I_{lim}$) is restricted to a few microamperes above the limiting current. These precautions are taken to mitigate potential degradation phenomena that could irreversibly alter the electrode interfaces [20]. Furthermore, gelatin is susceptible to thermal degradation induced by repetitive temperature cycles. In order to reduce this phenomenon, the upper temperature limit under operating conditions has been restricted to 310 K. Figure 5a,b presents the temperature-dependent profiles depicting the temporal evolution of voltage across the device in both the pristine and aged states, under bias currents of 20 μA and 7 μA, respectively. The employed logic driving circuit is shown in Figure 3a.

As depicted in Figure 5b, the aged temperature sensor maintains its ability to detect temperature variations within the range of 260 K to 310 K, comparable to its initial state. However, the ageing phenomena introduce degradation in the voltage response of the temperature sensor, evident in the discrepancies observed in voltage profiles across cycles at different temperatures. This effect becomes particularly pronounced at higher temperatures, notably at 310 K. It is likely associated with the temperature-dependent of the limiting current value, which increases with temperature. At 310 K, the $I_{bias}$ value closely approaches $I_{lim}$ (that is, 5 μA), prompting the sensor to switch to an ohmic regime. Specifically, the voltage signal exhibits a profile influenced of dissociation reactions within the device with the presence of a maximum or overshoot in the output voltage profile [49].

**Figure 6:**
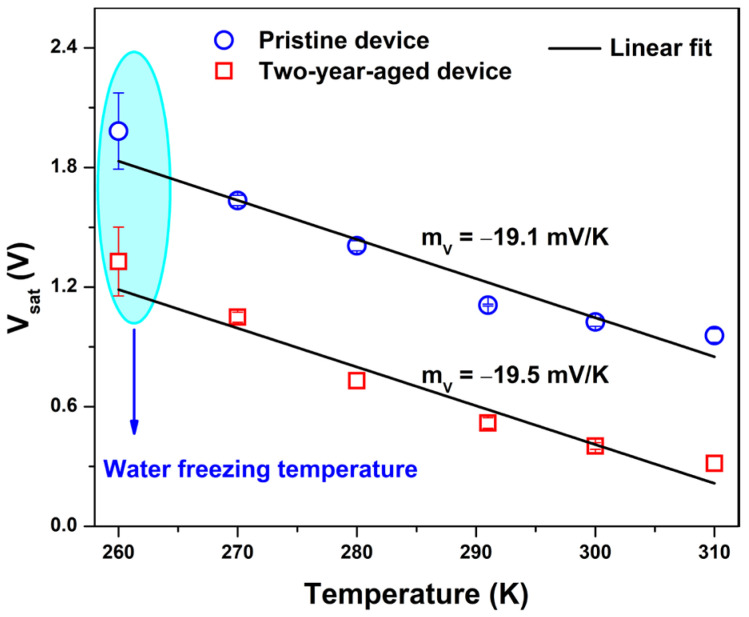
Comparison of the voltage measured across the sensor at the end of the sensing period $V_{sat}$ for both the pristine and aged states as a function of temperature. The solid line represents the linear fit.

In Figure 6, a comparative analysis of the voltage measured across the device at the end of the sensing period ($V_{sat}$) is presented for both the pristine and aged states as a function of temperature. As illustrated, the estimated sensitivity value $m_{V}$ of the temperature sensor (−19.5 mV/K) remains unaffected by ageing phenomena. This value, similar to that observed for the pristine device, surpasses that documented in the literature (approximately −11 mV/K) for a self-powered temperature sensor utilizing a gel-like electrolyte with a less environmentally friendly ionic liquid [15]. Furthermore, the aged sensor demonstrates continued capability in detecting ice formation. This finding is evident at 260 K, where the voltage level changes during each sensing cycle. This phenomenon can be ascribed to the freezing of water within the gel nanocomposite induced by subzero temperatures. Here, glycerol is able to form hydrogen bonds with water molecules, preventing the hydrogel from freezing. However, prolonged exposure to subzero temperatures results in increased rigidity of the blend, leading to a reduction in conductivity and subsequent modification of the output voltage profile compared to its initial cycle [50,51].

The shift in the sensitivity curve for the aged device over a two-year period, as depicted in Figure 6, resulting in the negative drift of the intercept, can be correlated with the modifications evident in the current–voltage characteristic presented in Figure 2. In this context, the ageing phenomena, attributed to the diffusion of water molecules out of the gel nanocomposite, have induced an augmentation in the overall resistance. Consequently, the bias current has been reduced from 20 μA to 7 μA, thereby giving rise to the observed offset in sensitivity.

Figure S2 (Supplementary Materials) illustrates a comparative analysis of the performance metrics of the environmentally temperature sensor, encompassing response time, power, and energy consumption during one hour of operation, in both its pristine and aged states. The electrochemical sensor, relying on ion assistance within the gelatin-based nanocomposite for its sensing mechanism, exhibits a rapid response time ∆τ (to attain the saturation value $\;∆\tau \approx 4\tau_{A}$) of 41.8 s to 28.9 s at 300 K for the pristine and aged states, respectively. Notably, the diminished response time in the aged device signifies a reduced participation of ions and water–glycerol molecules in dissociation reactions compared to the device in its initial state.

During the sensing mode implementation utilizing the logic circuit, the energy consumption for one operating hour (comprising six cycles) can be computed as $E\approx {6\cdot V}_{sense}\cdot I_{bias}{\cdot \tau }_{sense}$, where $I_{bias}$ is maintained at 20 μA (refer to Figure 2). Consequently, the resulting energy consumption is 8.1 μWh and 8.5 μWh at 300 K for the pristine and aged devices, respectively. Similarly, power consumption remains approximately 20 μW for both states, albeit with a slight increase observed in the aged device.

### 3.4. Regeneration Process of the Aged Device through Water Uptake

As widely reported in the literature, hydrogels exhibit exceptional dielectric and sensing properties attributable to their water content [20,24,52,53]. However, water within hydrogels is susceptible to evaporation in an open environment, particularly during temperature measurements. It is worth noting that the test structure of the examined device lacks complete encapsulation. The top and bottom electrodes are deposited on PET substrates, leaving a small area—corresponding to the perimeter of the sensor and the height of the hydrogel (approximately 1800 µm)—in contact with the open environment. Consequently, hydrogel nanocomposite may undergo a water loss, resulting in a deterioration of mechanical flexibility and electrical conductivity [20,52].

In our study, the investigated device demonstrates an overall increase in resistance, as detailed in Table 1, primarily attributed to the loss of water molecules. Despite this, the device proves capable of detecting temperature variations within the range of 260 K to 310 K, including ice formation. Notably, the performance metrics of the fabricated eco-friendly sensor (e.g., voltage sensitivity, time response, and energy consumption) align with those reported for the same temperature sensor in its pristine state, showcasing long-term stability. However, reproducibility and the shape of the output signal, in relation to the cycle number, are adversely affected, despite the implementation of the driving circuit. As the nanocomposite is fabricated within a heated water solution, it is possible to regenerate the electrical and mechanical properties of the aged sensor by remelting the blend and incorporating water through the exposed edge of the device. The gel nanocomposite, endowed with the capacity to absorb a volume of liquid water due to the presence of highly water-affinitive functional groups (e.g., –NH_2_, –OH, –COOH,), attains a hydrated state upon regeneration [53,54]. Precisely, the device can be subjected to heating at a temperature surpassing the gelation point and brought into contact with liquid water. Here, the gelation temperature ($T_{gel}$) of the hydrogel represents the threshold at which the substance attains the necessary conditions for gel formation [55,56]. 

**Figure 7:**
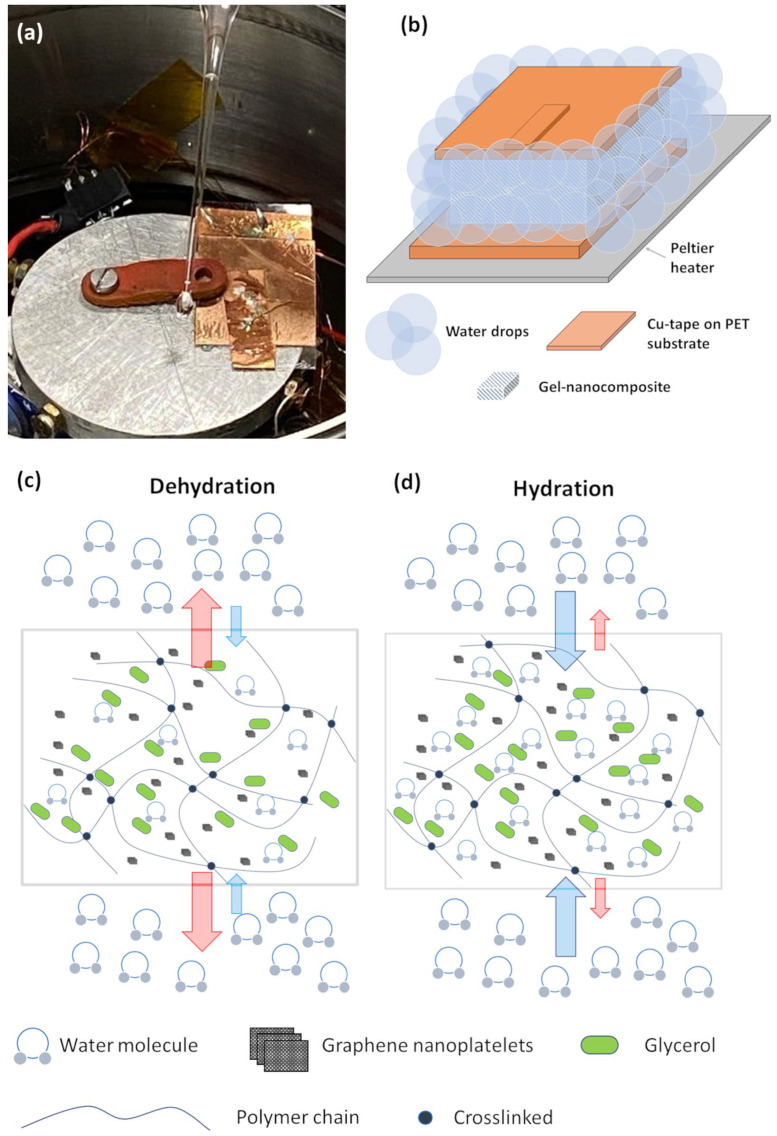
(**a**) Photograph and (**b**) schematic of the investigated eco-friendly sensor at 297 K before and during the hydration process consisting of depositing a few drops of water at the edge. Schematic representing the interactions between the water–glycerol molecules, the gelatin network, and the filler for the (**c**) aged (dehydrated state) and (**d**) regenerated (hydrated state) samples, respectively.

Figure 7a,b presents a photograph and the schematic of the investigated eco-friendly sensor at 297 K before and during the hydration process, respectively.

In this scenario, employing a gradually increasing temperature ramp, the gel nanocomposite transitions from a gel-like state to a liquid-like state, facilitating the infiltration of water drops inside the melted nanocomposite from the edge. The gel-sol transition in the context of hydrogels refers to the reversible phase transition between a gel state (a more solid or semi-solid state) and a sol state (soluble or liquid-like) [56,57]. This transition is influenced by environmental factors such as temperature, pH, or ionic strength [55,56,57].

Figure 7c,d illustrates a schematic depicting the interactions among water molecules, the gelatin network, the GNPs filler, and glycerol in the dehydrated state (aged device) and hydrated state after the regeneration. The aged device is marked by a desiccated hydrogel nanocomposite resulting from the outward diffusion of water molecules from the blend. This process alters the hydrogel network by inducing structural shrinkage, resulting in diminished mechanical flexibility and electrical conductivity, as shown in Figure 7c. While ideally, the hydrogel should absorb humidity from the ambient environment, the prevailing trend is the dominant outward diffusion of water [24]. The diffusion of water molecules out of the blend and the segregation of additives, such as glycerol and fillers, are the main mechanisms of the degradation of the eco-friendly devices fabricated with biopolymer over time [24,45,58].

It is noteworthy that through the regeneration process, the blend tends to expel water from the melted matrix (see red arrows). Simultaneously, the water deposited along the edge compensates for the diffusion out of the hydrogel, ultimately hydrating the resultant gel nanocomposite. The regeneration process causes a swollen of the polymer matrix that absorb the water [59]. To investigate the thermal stability of the hydrogel throughout operational cycles the electrical resistance of the device has been monitored at varying temperatures. It is well established that the thermal characteristics of hydrogels (e.g., thermal stability, thermoresponsiveness, and thermal conductivity) can significantly influence the performance of electronic devices [52].

**Figure 8:**
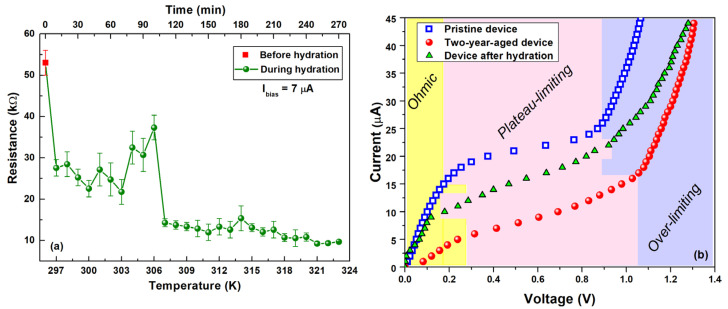
(**a**) Resistance evolution in temperature (bottom x-axis) and time (top x-axis) during the hydration of the aged eco-friendly temperature sensor. A resistance peak is visible at the gelling temperature (306 K), while a pronounced reduction is observed for elevated temperatures. (**b**) Current–voltage characteristics of the pristine, two-year-aged, and hydrated temperature sensor under current biasing. The colored regions are shifted following the different working conditions of pristine, hydrated, and aged device, respectively.

In Figure 8a, the resistance evolution in the temperature ranges between 297 K and 323 K (bottom x-axis) and in time (top x-axis) is shown. To facilitate the hydration of the hydrogel during the temperature ramp, the aged device is left at each temperature for approximately 10 min. Here, a resistance peak is visible at a temperature of about 306 K, while a pronounced reduction is observed for elevated temperatures. This value corresponds to the gelling temperature $T_{gel}$ and the presence of an overshoot indicates the occurring of the phase transition in the blend. The aged device exhibits an initial resistance value (~54 kΩ), consistent with that reported in Figure 2. As the temperature continues to rise, a progressive decline in resistance occurs, culminating in a peak at 37 kΩ at 306 K ($T_{gel}$). Subsequently, beyond $T_{gel}$, the recorded resistance stabilizes at approximately 14 kΩ, gradually diminishing with decreasing temperature to 9 kΩ at 323 K. At this final stage, following a 3-h measurement period, the water around the perimeter vanishes, with a portion being absorbed and another undergoing evaporation. The gelling temperature of the hydrogel can be influenced by several factors: composition of the gel, cross-linking density, polymer concentration, solvent properties, presence of additives, temperature ramp rate, and hydration conditions [8,53,56]. Gel nanocomposite formulated with gelatin, GNP, and a water–glycerol matrix demonstrate a $T_{gel}$ within the range of 303 K (30 °C) to 313 K (40 °C) [56,57,60], consistent with the findings illustrated in Figure 8a. Notably, electrical measurements have proven effective in determining $T_{gel}$ values, exhibiting comparability with well-established techniques such as rheological analysis, viscosity measurements, and thermal conductivity characterizations [55,56,57,61]. This feature has been already observed in the literature by monitoring the electrical characteristics of several devices (e.g., solar cells) [37].

In Figure 8b, a comparative analysis of the current–voltage characteristics is presented for the pristine temperature sensor, aged over a two-year period, and the same sensor subsequent to hydration under current biasing. Notably, the current–voltage characteristic of the regenerated sensor exhibits a shift towards a higher current range after hydration. This transition aligns with the observed increase in the limiting current value following water absorption. As illustrated in Figure S3 (Supplementary Materials), the limiting current $I_{lim}$ attains a value of 8 μA for the hydrated device, surpassing that reported for the aged device (5 μA). The increased value of $I_{lim}$ after the regeneration procedure combined with the shift in the electrical characteristic indicates the beneficial impact of water molecule absorption within the gel nanocomposite during the hydration process, resulting in an overall reduction in electrical resistance. These findings suggest that the temperature sensor is regenerated after two years.

## 4. Conclusions

A water-processable and cost-effective nanocomposite material, comprising gelatin and graphene, has been utilized for the development of an environmentally friendly temperature sensor. The sensor demonstrates prolonged stability over a two-year period, maintaining a consistent temperature voltage sensitivity of about −19 mV/K within the range of 260 to 310 K and the capability to detect ice formation. The aged sensor shows an overall increase in electrical resistance attributed primarily to the loss of water molecules. Nevertheless, dissociation reactions within the sensor, which cause limiting-current phenomena in the gelatin nanocomposite, are still present. Here, the dehydration process of the device results in a significant reduction in the ion-conducting path, consequently leading to an increase in resistivity and a corresponding decrease in the limiting current value. This reduction is correlated with the diminished concentration of available water–glycerol molecules involved in dissociation reactions, leading to an observed shift in the electrical characteristics toward the low current region. Despite these changes, the performance metrics of the eco-friendly sensor, including voltage sensitivity, time response, and energy consumption, remain comparable to those reported for the sensor in its pristine state. The electrochemical sensor demonstrates a rapid response time of 41.8 s and 28.9 s at 300 K for the pristine and aged states, respectively. During the sensing mode implementation with the driving circuit, the resulting energy consumption is 8.1 μWh and 8.5 μWh at 300 K for the pristine and aged devices, respectively, with power consumption remaining approximately 20 μW for both states, showcasing a long-term stability.

A detailed model describing charge carrier accumulation, faradaic charge transfer, and diffusion processes under current-controlled conditions has been proposed. Here, the aged temperature sensor sustains low power consumption in the microwatt range and exhibits partial self-powering capability, generating approximately 30% of the required energy for its operational needs.

The thermal stability of the gel nanocomposite in the range between 297 K and 323 K has been evaluated. Experimental evidence confirms the efficient regeneration of aged temperature sensor by infiltrating of water at a temperature higher than the gelation point of the gel nanocomposite. Real-time monitoring during the hydration procedure reveals that the regeneration process initiates at the gelation point (~306 K), resulting in a more conductive nanocomposite. Following hydration, the regenerated sensor displays a significant shift in the current–voltage characteristic towards a higher current range, aligned with an observed increase in the limiting current value. This increased $I_{lim}$ value, coupled with the shift in electrical characteristic, underscores the advantageous impact of water molecule absorption within the gel nanocomposite during the hydration process. A distinct regeneration of the low-power and eco-friendly temperature sensor is evident after two years. These characteristics render the electrochemical temperature sensor well-suited for energy-efficient applications, such as in indoor monitoring scenarios aimed at optimizing the energy performance of buildings.

## Figures and Tables

**Table 1:** Best fitting values of the parameters in Equation (1) for the pristine and aged devices.

Device State	*R_S_* (kΩ)	*R_A_* (kΩ)	*C_A_* (mF)	*R_B_* (kΩ)	*C_B_* (mF)	*V*_0_ (V)
Pristine	14.17	9.64	1.08	108.74	0.50	0.26
Aged	16.66	26.39	0.27	45.59	1.96	0.32

## Data Availability

The data presented in this study are available on request from the corresponding author.

## Supplementary Materials

The following supporting information can be downloaded at: https://www.mdpi.com/article/10.3390/nano14030283/s1, Figure S1: Determination of the limiting current value by using the method of Cowan and Brown for the (a) pristine and (b) aged temperature sensor, respectively; Figure S2: Radar plot comparing the performance of the eco-friendly temperature sensor in the pristine and aged states, respectively; Figure S3: Determination of the limiting current value by using the method of Cowan and Brown for the device after hydration.
