# Supplementary material for: Regeneration and Long-Term Stability of a Low-Power Eco-Friendly Temperature Sensor Based on a Hydrogel Nanocomposite
Source: Nanomaterials (Basel). 2024 Jan 30;14(3):283. doi: 10.3390/nano14030283 (PMC10856540; doi:10.3390/nano14030283)
Supplement: Supplementary file 1 [file nanomaterials-14-00283-s001.zip › nanomaterials-2811863-supplementary.pdf]

nanomaterials MDPI

Supplementary materials: Regeneration and Long-Term Stability of Low-Power Eco-Friendly Temperature Sensor Based on Hydrogel Nano-composite

Giovanni Landi [1], Sergio Pagano [2,3,4,*], Veronica Granata [2,3], Guerino Avallone [2,3], Luca La Notte [5], Alessandro Lorenzo Palma [5], Paolo Sdringola [5], Giovanni Puglisi [5] and Carlo Barone [2,3,4,*]

1 ENEA Centro Ricerche Portici, Piazzale Enrico Fermi, Località Granatello, 80055 Portici, Italy; giovanni.landi@enea.it
2 Dipartimento di Fisica "E.R. Caianiello", Università degli Studi di Salerno, 84084 Fisciano, Salerno, Italy; spagano@unisa.it (S.P.); vgranata@unisa.it (V.G.); guavallone@unisa.it (G.A.); cbarone@unisa.it (C.B.)
3 INFN Gruppo Collegato di Salerno, c/o Università degli Studi di Salerno, 84084 Fisciano, Salerno, Italy;
4 CNR-SPIN, c/o Università degli Studi di Salerno, 84084 Fisciano, Salerno, Italy;
5 ENEA, Casaccia Research Center, Via Anguillarese 301, 00123 Rome, Italy; luca.lanotte@enea.it (L.L.N.); alessandrolorenzo.palma@enea.it (A.L.P.); paolo.sdringola@enea.it (P.S.); giovanni.puglisi@enea.it (G.P.)
* Correspondence: spagano@unisa.it (S.P.); cbarone@unisa.it (C.B.)

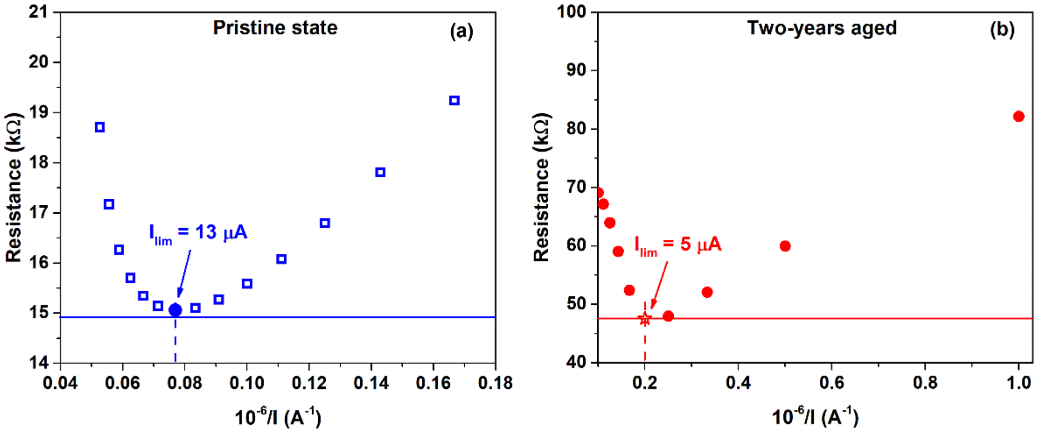

Figure S1. Determination of the limiting current value by using the method of Cowan and Brown for the (a) pristine and (b) aged temperature sensor, respectively.

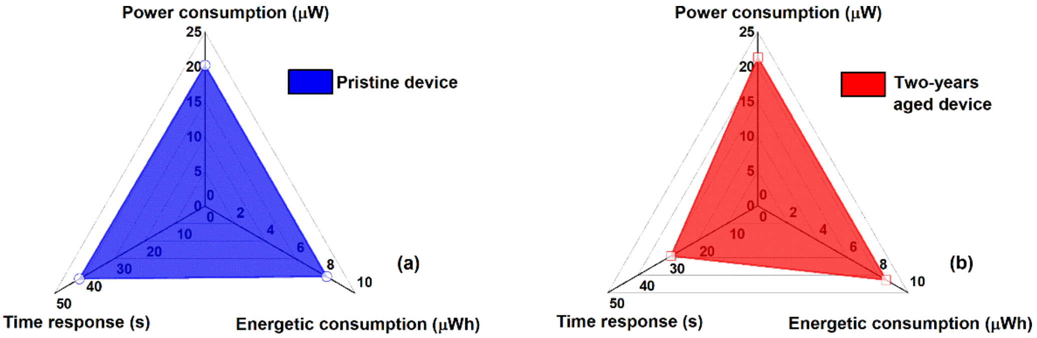

Figure S2. Radar plot comparing the performance of the eco-friendly temperature sensor in the pristine and aged states, respectively.

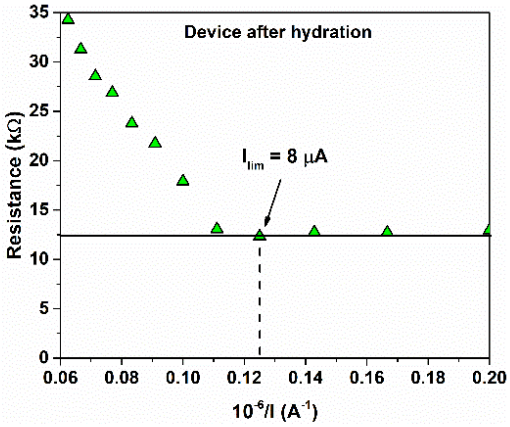

Figure S3. Determination of the limiting current value by using the method of Cowan and Brown for the device after hydration.
